# Vick’s Floral Guide, 1894

**Published:** 1894-03

**Authors:** 


Vick's Floral Guide, 1894.

It contains descriptions that describe, not mislead; illustrations that instruct, not exaggerate. This year it comes to us in a suit of gold. Printed in eight different colors beside black. Colored plates of Chrysanthemums, Poppies, and vegetables. On the front cover is a very exquisite bunch of Vick's New White Branching Aster, and on the back is the New Double

Anemone; one hundred and twelve pages filled with many new novelties of value as well as all the old leading varieties of flowers and vegetables.

We advise our friends who intend doing anything in the garden this year to consult Vick before starting operations. Send 10 cents to James Vick's Sons, Rochester, N. Y., for *Vick's Guide*, it costs nothing, as you can deduct the 10 cents from first order. It certainly will pay you.

